# Validation of a web-based dietary assessment program against 24-h recalls in adults with type 1 diabetes

**DOI:** 10.3389/fnut.2024.1395252

**Published:** 2024-11-27

**Authors:** Afroditi Alexandra Barouti, Stephanie Erika Bonn, Anneli Björklund

**Affiliations:** ^1^Department of Molecular Medicine and Surgery, Karolinska Institutet, Stockholm, Sweden; ^2^Center for Diabetes, Academic Specialist Center, Stockholm, Sweden; ^3^Division of Clinical Epidemiology, Department of Medicine Solna, Karolinska Institutet, Stockholm, Sweden

**Keywords:** validity, web-based dietary records, nutrition methodology, diet registration, dietary assessment, 24-h dietary recall, type 1 diabetes, carbohydrate counting

## Abstract

**Background:**

Nutrition Data is a web-based program for nutrition analysis and registration of diet and exercise. It may aid dietary assessment and carbohydrate counting in people with type 1 diabetes (T1D) but requires validation.

**Objectives:**

To assess relative validity of Nutrition Data in measuring energy, carbohydrate and other macronutrient intake and evaluate the program’s user acceptability, in adults with T1D.

**Methods:**

In this validation study, we analyzed data from 42 participants (median age 46.5 years, 45% women) from the DANCE study, a randomized controlled trial comprising of individuals with T1D in Sweden. Mean intakes of energy, carbohydrates, fat, protein, alcohol, fiber, sugars and saturated fat from 2 days registered in Nutrition Data were compared against the respective intakes acquired by unannounced 24-h-recalls of the same days. Paired sample *t*-tests and Wilcoxon matched-pairs signed rank tests were used to compare mean intakes between the two methods, and Spearman’s rank correlation and Bland Altman plots were used to assess agreement between the methods. Usability and user acceptability of Nutrition Data were assessed with a questionnaire.

**Results:**

There were no significant differences in mean dietary intakes between the two methods. Spearman’s correlation coefficients ranged from *r* = 0.79 for energy intake to *r* = 0.94 for carbohydrate intake (% total energy intake) (*p* < 0.001 for all outcomes). The Bland–Altman plots showed no clear patterns of bias, though limits of agreement were relatively wide. Most participants found Nutrition Data easy to use (70%), helpful for carbohydrate counting (88%) and would recommend it to others (73%).

**Conclusion:**

The web-based program Nutrition Data showed good validity in assessing intake of energy and macronutrients compared to 24-h recalls and high user acceptability in Swedish men and women with T1D, and could, therefore, be used to facilitate diet registration and carbohydrate counting.

## 1 Introduction

Nutritional therapy is essential for achieving glycemic, metabolic and nutritional goals for all people with diabetes (1). Assessing food intake in a valid and time-effective way is needed when treating patients with diabetes, as well as in research to ensure the quality of nutritional interventions (2). For people with type 1 diabetes (T1D), accurately estimating the amounts of carbohydrates in meals to adjust meal insulin doses is an important part of treatment that has been associated with increased flexibility and improved glycemic control (1, 3). Traditionally, dietary intake data is collected through subjective methods, such as paper-based food diaries and 24-h dietary recalls (24HRs) (4, 5). However, these methods require additional administration time from health care professionals for nutritional analysis and may be burdensome for patients (4, 5).

Nowadays, there are innovative technologies used for dietary assessment, such as web-based programs and mobile apps, which have the benefit of being more time- and cost-effective (6, 7). However, these are not without limitations, as they entail considerable costs for development, modification, and maintenance, and require technological literacy by users (6, 7). To our knowledge, technologies specifically developed and validated for people with T1D that allow simultaneous registration of diet, physical activity, blood sugar, and insulin doses, are not currently available.

The web-based program Nutrition Data Sweden (from here on written as “Nutrition Data”), with added features on diabetes-specific information, was used within the randomized controlled trial DANCE to assess dietary intake in adults with T1D. The DANCE study aimed to compare how diets with different amounts of carbohydrates affect insulin requirements, metabolic control, and glycemic variability. Nutrition Data also has the potential to be used at diabetes clinics as a time-efficient alternative to 24HRs. However, to ensure the quality of the collected dietary information, the program needs to be validated.

The primary aim of this study was to evaluate the relative validity of Nutrition Data in measuring energy and macronutrient intake compared to intakes derived using more traditional 24HRs in adults with type 1 diabetes. Secondly, we aimed to assess the usability and user acceptability of Nutrition Data within the same population.

## 2 Materials and methods

### 2.1 Study sample

Participants recruited for this validation study were a subsample of men and women with T1D who participated in the DANCE study in the Stockholm and Uppsala Region of Sweden. In brief, the Diabetes ANd CarbohydratEs study (DANCE) is an ongoing randomized controlled trial with three arms, in which adults with T1D are randomized to diets with different amounts of carbohydrates (low, moderate or high intake) during 6 months (intervention period). Participants are thereafter followed for an additional 6-month period, during which they can eat a diet of their choice. Inclusion criteria for the DANCE study were age ≥ 20 years and T1D duration of at least 1 year, and exclusion criteria were BMI <18.5 kg/m^2^, c-peptide ≥0.3 nmoL/L, serious cardiovascular or kidney disease, hepatic failure, ongoing or planed pregnancy or lactation, alcohol/drug problems, eating disorders, serious hypoglycemia unawareness and if participants did not plan to follow one of the randomized diets. The DANCE study is registered at clinicaltrials.gov, registration number: NCT03761186.

For the purposes of this validation study, half of the participants that were asked and agreed to participate were in the first 6 months of the DANCE study (intervention phase) and half were in the last 6 months of the study. Those that were in the first 6 months were asked to participate after they had completed 6 weeks of follow-up so that they had enough time to learn registering in Nutrition Data. Invited participants were informed that participation in the validation study would not affect their participation in the DANCE study.

The current validation study was conducted according to the guidelines of the Declaration of Helsinki and written informed consent specific for the validation study was obtained from all participants. The participants of the validation study received a flower gift card to thank them for their participation. The study has been approved by the Regional Ethics Review Board of Stockholm (2018/218-31, 2018/2349-32, 2019-02315, 2020-04116, and 2023-01789-02).

### 2.2 Web-based program for diet registration

**Figure 1:**
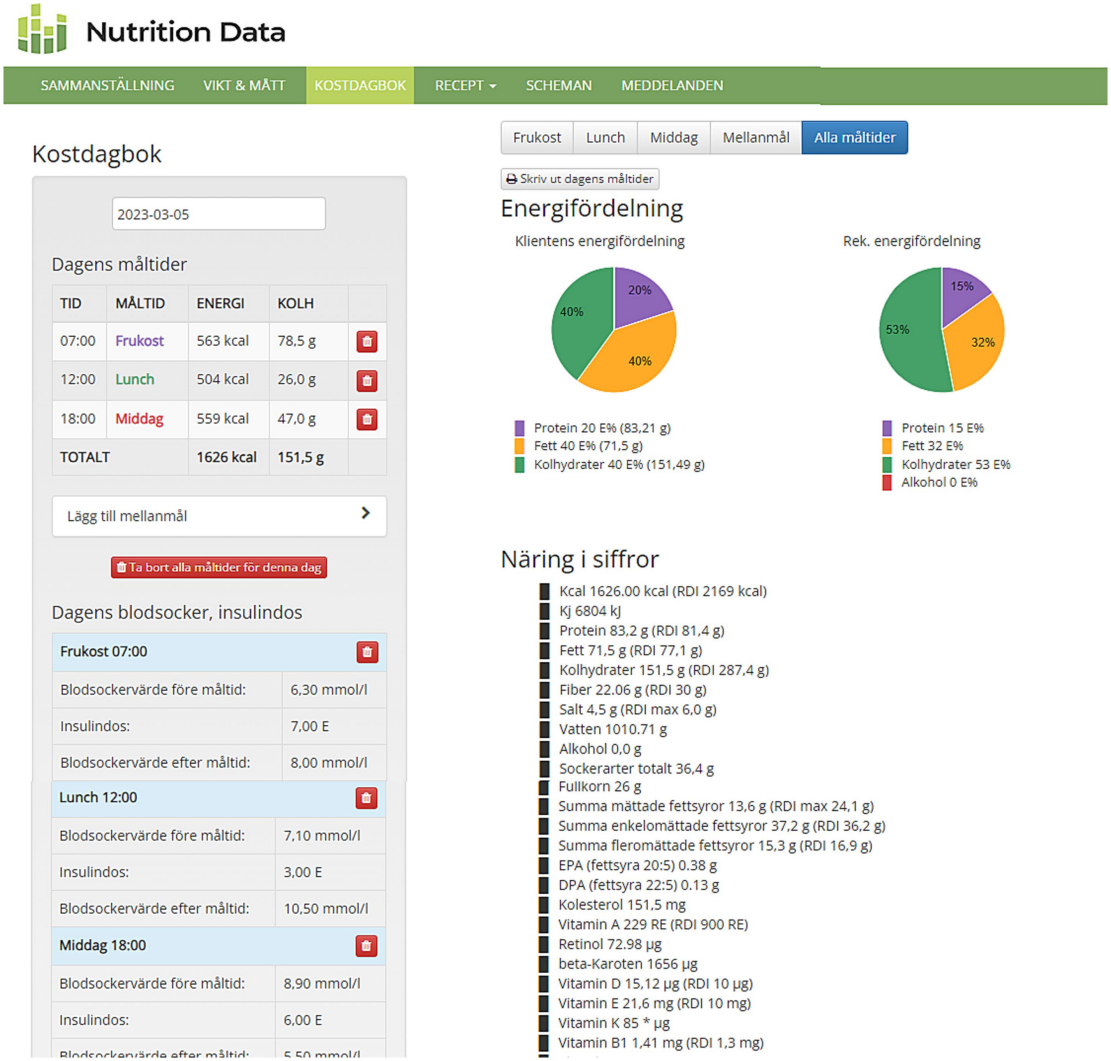
Screenshot from Nutrition Data user view. On the left side are the registered meals of the day, with energy intake in kcal and carbohydrate intake in grams shown per meal and total per day. Below on the left side are the registered pre- and postprandial blood sugar values (mmol/l) and meal insulin doses (IU). On the right side are the diagrams of energy distribution from each macronutrient (shown with different colors); the first pie chart on the left is the user’s actual energy distribution for the day while the second one on the right is the administrator’s or researcher’s recommendation for energy distribution for this user. Below the pie charts are daily intakes in grams for different macro- and micronutrients.

Nutrition Data is an already developed web-based program and mobile app for nutritional analysis, as well as diet and exercise tracking (8). Nutrition Data was upgraded before the DANCE study start to include features specifically designed for the study, including information on carbohydrate amounts per food item and meal to aid carbohydrate counting, diagrams of participants’ actual energy distribution from the different macronutrients as well as the recommended distribution according to the randomized diet, and entries for registering pre- and postmeal blood sugar levels and insulin doses. The version of Nutrition Data used in the DANCE study was web-based and could be used on any device connected to the Internet, including smartphones as it had a mobile friendly view. A screenshot from Nutrition Data user view is shown in Figure 1.

Nutrition Data also includes a researcher/dietitian administrative platform for creating and managing participants’ accounts and setting nutritional goals, remotely accessing and assessing users’ registered data (no data transfer or synchronization is required) and creating detailed nutritional analysis reports. Nutrition Data is linked to the National Food Database of Sweden and is supplemented by foods from the Finnish, Norwegian and American food databases that are relevant for the Swedish market (9–12).

When users register their food and beverage intake in the program, they start by choosing a date and then add a meal or snack. They then use the search function to select the food items they want to consume from the program’s food database, and lastly, they define their portion size for each food item by entering the consumed quantity (in g, mL, portions if defined portion sizes are available, or household measures, e.g., dL, tsp., tbsp). Users can also pick from “recently used items” to register repeated foods/drinks and they can save their “favorite meals” for easy retrieval and registration on a different day or meal occasion. In case participants cannot find a specific food item in the database, they can either choose an alternative item with similar nutritional content, which is shown when browsing through items, or write a note about the missing food item in the notes section of that day. The dietitian can then review the notes and aid with registration. The program also allows the user to create and save recipes or choose from a list of nutritionally calculated recipes, which can be sorted by, e.g., main ingredient, type of diet, energy or carbohydrate intake. Photos of meals can also be uploaded. In order for users to track their intake and follow the randomized diet as closely as possible, macro- and micronutrient intake as well as diagrams with the energy distribution from macronutrients are shown for each meal and day. Users can also record physical activity by choosing different activities from the program’s database and registering time and duration.

### 2.3 Study design

In this validation study, energy and macronutrient intakes obtained from web-based diet registration in Nutrition Data were compared to the intakes obtained from 24HRs. 24HRs were chosen as the comparison method because it is the most commonly used method to collect dietary information from patients with diabetes at the clinic where the study was performed as well as in other clinical settings.

**Figure 2:**
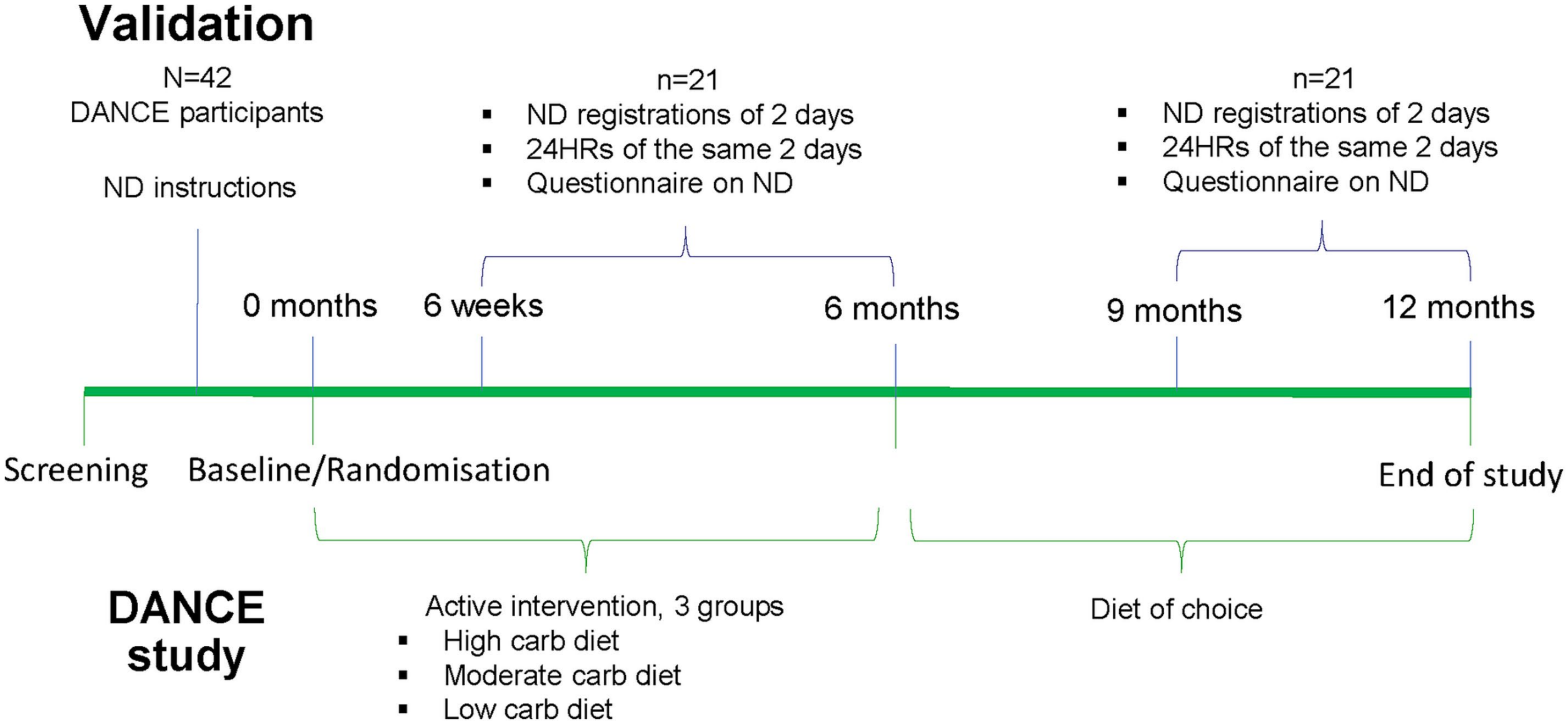
Study design of the validation study performed within the randomized controlled trial DANCE. ND, Nutrition Data.

The design of the validation study, which is performed within the DANCE study, is shown in Figure 2. It should be noted that the validation study did not have a randomized design. Participants of the DANCE study got an individual review of the program’s functions and user instructions before study start and registered their food intake in Nutrition Data regularly, with a minimum requirement of four registered days before every follow-up visit at 3- and 6 weeks, and 3-, 6-, 9-, and 12 months.

For the validation, a trained study dietitian conducted two 24HRs per participant on two of the days the participants already had registered their food intake in Nutrition Data before a planned study visit. Usually, one recall was performed at the clinic the day of the first planned study visit and the other one was performed by a phone call in order to reduce participant burden of extra study contacts. The first 24HR was always unannounced. As for the second, the participants were informed that it would be performed on one of the remaining 3 days of that diet registration period or on a day during the next one. The day of the second recall was chosen by study personnel to ensure that data included one weekday and one weekend day, i.e., if the first recall happened to be a weekday, the second one was a weekend day, and vice versa. During the 24HRs, participants had access to the Swedish Food Agency’s Portion Guide, a booklet with portion size pictures of common food items and were prompted to use that or household measurements for describing their consumed portions (13). The 24HR data were then entered by the study dietitian in Nutrition Data’s nutritional analysis feature, which calculated energy and macronutrient intakes per day and participant.

The following intakes were assessed and compared between participants’ own registrations in Nutrition Data and the 24HRs: energy (kcal/day), carbohydrate as a percentage of total energy intake (% TEI) and as absolute amounts (g/day), fat (% TEI and g/day), protein (% TEI and g/day), alcohol (% TEI and g/day), fiber (g/day), sugars (g/day) and saturated fat (g/day).

After having completed both 24HRs, each participant filled out a written 18-item questionnaire assessing usability and user acceptability of Nutrition Data, details of which are presented in the Supplementary material. This questionnaire was an adaption of other existing mHealth satisfaction questionnaires (14, 15), and specifically the version by Melin et al. (15), which has been evaluated within an adult population in Sweden using Rasch measurement theory.

### 2.4 Power calculation

Power analysis for the comparison of mean intake values between the two methods (Nutrition Data vs. 24HRs) was performed using carbohydrate intake (% of TEI) as main outcome since this was the randomization variable in the DANCE study. A difference of ≥6% in carbohydrate intake (% of TEI) between the two methods was considered relevant for validation purposes, i.e., the methods would then differ significantly. With a standard deviation in carbohydrate intake of 7% according to the latest national survey of the Swedish Food Agency for adults in Sweden (16), a significance level of 0.05 and a power of 0.80, a total of n = 42 participants was needed to find significant differences between the methods if these existed.

### 2.5 Statistical analysis

Descriptive statistics were used to present the baseline characteristics of the study participants and user acceptability of Nutrition Data. Continuous variables are presented as means with standard deviations (SD) if normally distributed, or medians with interquartile ranges (IQR) if skewed. Categorical variables are presented as number and percentages. Differences between mean intake of the 2 days estimated by Nutrition Data and 24HR were assessed with paired samples *t*-test for normally distributed data otherwise Wilcoxon matched-pairs signed-rank test for non-parametric data. Spearman’s rank correlation coefficients were used to assess the relationship in estimating energy and macronutrient intake between the two methods. The Bland–Altman method (17) was used to assess the agreement between the two methods. Specifically, the difference between the methods was plotted on the y-axis (intake measured by Nutrition Data – intake measured by 24HRs) against the average intake estimated by the two methods on the x-axis. Mean difference as well as the limits of agreement (±2SD) were calculated and presented in the Bland–Altman plots. Supplementary analyses investigated whether the differences between the two methods (Nutrition Data-24HRs) differed between study periods (intervention time 0–6 months vs. diet of choice 6–12 months) using *t*-test or Wilcoxon rank-sum test for unmatched data. Correlation analyses were also repeated separately for each study period. Finally, the quality of the dietary assessment was evaluated based on the checklist developed by Wang et al. (2). *p* < 0.05 was considered significant. Analyses were performed with STATA/IC Version 16.1 (StataCorp, College Station, Texas, USA).

## 3 Results

Out of 44 contacted participants in the DANCE study, 42 agreed to participate in the validation study. Table 1 shows the baseline characteristics of the study participants.

**Table 1:** Baseline characteristics of study population (*n* = 42).

Age (years)	46.5 (35, 59)
Sex (females)	19 (45%)
BMI (kg/m^2^)	26.4 ± 4.5
Type 1 diabetes duration (years)	21 (9, 28)
Pump users	17 (40%)
Highest level of education	
High school	9 (21%)
University	26 (62%)
Vocational training	7 (17%)

Data are presented as numbers (percentages), medians (interquartile range) or means (standard deviation).

Table 2 presents the mean intakes for energy and macronutrients assessed using Nutrition Data and 24HR. Nutrition Data estimates of mean energy and nutrient intakes were very similar compared to the 24HR estimates, in general 0–3% lower for all nutrients except for mean protein intake that was 1% higher, and not statistically significant. Table 3 shows the correlations between Nutrition Data and 24HR for all outcomes. There were statistically significant correlations ranging from 0.79 for energy intake (kcal/day) to 0.94 for carbohydrate intake (g/day) (*p* < 0.001 for all correlations).

**Table 2:** Mean intake (± SD) estimated by means of Nutrition Data registrations and 24-h recalls (*n* = 42).

Intake	Nutrition Data food diary	24-h recall	*P*-value
Energy (kcal/day)	1784 ± 430	1818 ± 422	0.33
Carbohydrate (%TEI)^a^	35 ± 10	35 ± 9	0.72
Fat (%TEI)^a^	45 ± 10	46 ± 9	0.40
Protein (%TEI)	18 ± 4	18 ± 5	0.38
Alcohol (%TEI)	1 ± 2	1 ± 2	0.62
Carbohydrate (g/day)	148 ± 50	151 ± 51	0.29
Sugars (g/day)	46 ± 22	47 ± 22	0.64
Fiber (g/day)^a^	21 ± 8	21 ± 7	0.80
Fat (g/day)	91 ± 34	94 ± 32	0.42
Saturated fat (g/day)	30 ± 12	31 ± 15	0.61
Protein (g/day)	81 ± 25	80 ± 26	0.87
Alcohol (g/day)	2 ± 5	2 ± 5	0.76

^aNormally distributed variables. Differences between the two methods were assessed by paired t-test for normally distributed variables otherwise Wilcoxon matched-pairs signed-rank test.^

**Table 3:** Correlations between Nutrition Data registrations and 24-h recalls (*n* = 42).

	Spearman’s rho
Energy kcal/day	0.79
Carbohydrates % TEI	0.94
Carbohydrates g/day	0.93
Of which, sugars g/day	0.87
Fibre g/day	0.83
Fat %TEI	0.90
Fat g/day	0.85
Saturated fat g/day	0.89
Protein % TEI	0.84
Protein g/day	0.86
Alcohol %TEI	0.88
Alcohol g/day	0.90

*P < 0.001 for all coefficients.*

**Figure 3:**
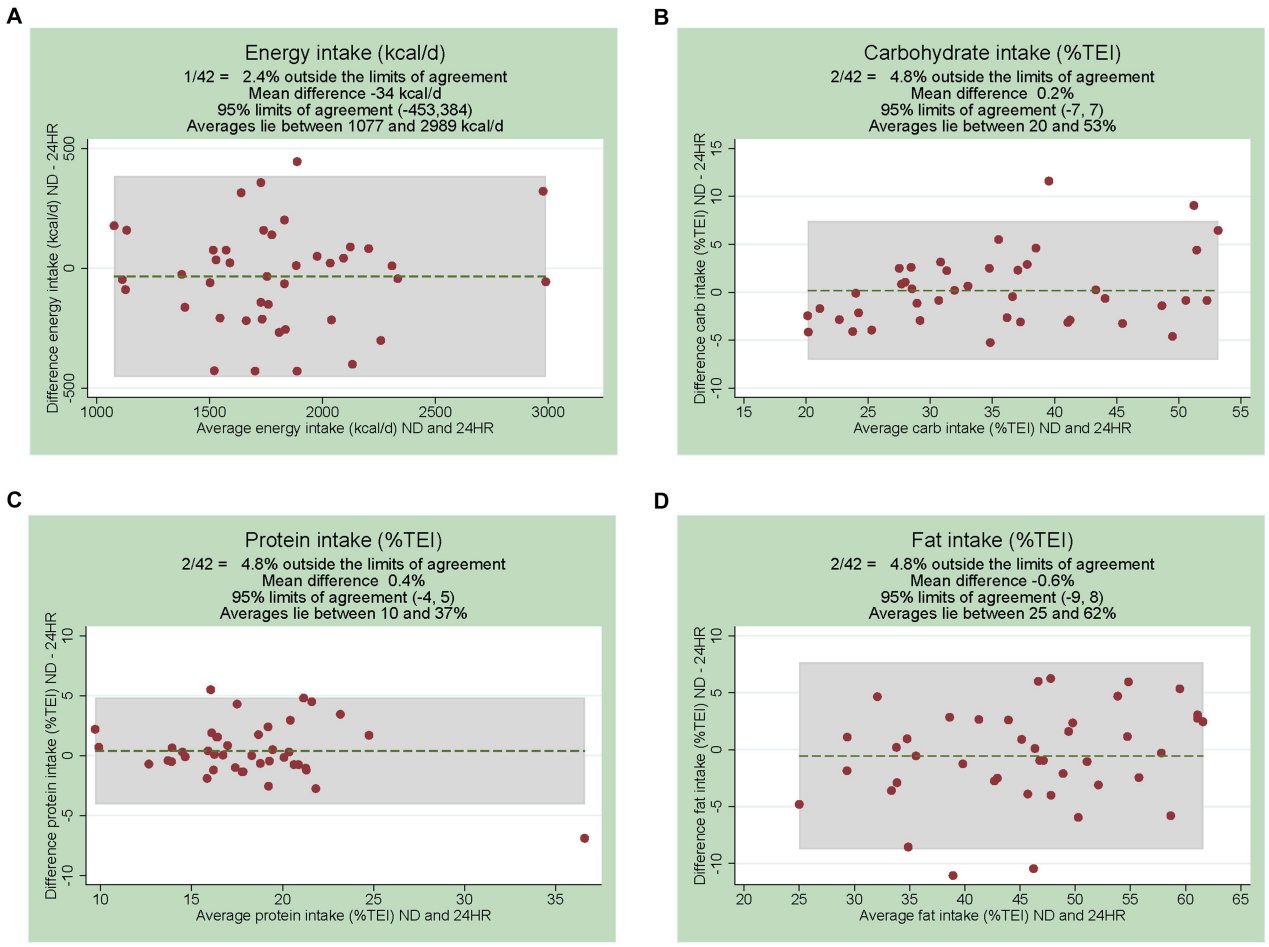
Bland Altman plots showing the difference of the two methods on the y-axis (ND-24HR) and the average of the two methods on the x-axis. Intakes shown: **(A)** energy (kcal/day), **(B)** carbohydrate (% of total energy intake, TEI), **(C)** protein (%TEI) and **(D)** fat (%TEI). *ND*; Nutrition Data. 24HRs, 24-h recalls.

Figure 3 shows the Bland Altman plots for the agreement between the Nutrition Data registrations and 24HRs in assessing intake of energy and carbohydrates, fat and proteins in % of TEI. For energy intake, the mean difference was 34 kcal/day less for Nutrition Data compared to 24HRs (−1.9%) with wide 95% limits of agreement defined as ±2SDs (−453, 384 kcal/day), though the differences of the two methods varied unsystematically across the different average energy intakes. Wide limits of agreement suggest that while the program and 24HRs are generally consistent, individual discrepancies may still exist in certain cases. For carbohydrate intake (% TEI) the difference between the two methods was more often negative, i.e., higher intakes were reported by 24HRs compared to Nutrition Data, across the lower and higher average carbohydrate intakes. However, this trend was not seen when carbohydrate intake was measured in g/day, with limits of agreement ranging from −39 to +33 g/day (Supplementary Figure 1). The Bland–Altman plots for the remaining intakes (protein and fat in g/day, saturated fat g/day, sugars g/day, alcohol %TEI and g/day) showed no clear patterns of bias except for fiber intake (Supplementary Figure 1); as average fiber intakes increased, Nutrition Data registrations reported higher intakes compared to the 24HRs.

In secondary analyses, we investigated whether differences in measuring mean intakes between the two methods (Nutrition Data−24HRs) were different between participants following specific diets during intervention period in DANCE study (0–6 months) compared to participants following their diet of choice (6–12 months). There were no significant differences between the two study periods (Supplementary Table 1). Correlation analyses for the two methods were repeated separately for each study period (Supplementary Table 2), with the Spearman correlation coefficient ranging from 0.78 to 0.99 for the 0–6 months study period (*p* < 0.001 for all outcomes) and from 0.78 to 0.95 for the 6–12 months study period (*p* < 0.001 for all outcomes). The quality of the dietary assessment was evaluated as medium with a score of 5 out 8 points (Supplementary Table 3).

Table 4 shows results of user acceptability and usability of Nutrition Data. Two participants did not return a completed questionnaire and one responded only to the first 14 questions. The majority of the participants found Nutrition Data easy to use (70%), helpful for carbohydrate counting (88%) and would recommend it to others (73%). However, 25% disagreed that it was easy to find the correct food item to register and 38% were neutral to this statement. One third of the participants used Nutrition Data to count carbohydrates and take meal insulin thereafter sometimes and 36% used it for this purpose most or all the time. Of the 39 participants that responded to the 16^th^ question, 33 (85%) would register their food intake in Nutrition Data just before, during or just after a meal and 6 (15%) would register at the end of the day or the next day.

**Table 4:** User acceptability and usability of Nutrition Data (*n* = 40, 2 missing).

	Disagree (score 1 + 2)	Neutral (score 3)	Agree (score 4 + 5)
	*n* (%)	*n* (%)	*n* (%)
Easy to use	1 (2.5)	11 (27.5)	28 (70.0)
Fun to use	4 (10.0)	9 (22.5)	27 (67.5)
Time spent for ND was well-used	1 (2.5)	5 (12.5)	34 (85.0)
Difficult to remember using ND	22 (55.0)	11 (27.5)	7 (17.5)
The user instructions given were enough	0 (0)	2 (5.0)	38 (95.0)
Too time consuming	16 (40.0)	13 (32.5)	11 (27.5)
Disturbed everyday life	29 (72.5)	5 (12.5)	6 (15.0)
Was boring to use	33 (82.5)	4 (10.0)	3 (7.5)
Would recommend it	3 (7.5)	8 (20.0)	29 (72.5)
Would prefer a paper-based food record	35 (87.5)	3 (7.5)	2 (5.0)
Easy to find the correct food-item to register	10 (25.0)	15 (37.5)	15 (37.5)
Easy to find and register correct portion size	6 (15.0)	8 (20.0)	26 (65.0)
Helped estimate carbohydrate amount	2 (5.0)	3 (7.5)	35 (87.5)
Helped follow the randomised diet	0 (0)	8 (20.0)	32 (80.0)

Participants estimated that they spent a median time of 19 (IQR 10, 30) min/day registering their daily intake in Nutrition Data. In the last open question, some of the program’s strengths mentioned multiple times were the comprehensive information on carbohydrate intake (per food item, meal and day) that users receive when they register, and the program’s diagrams, e.g., the pie charts for energy distribution among macronutrients. Among the perceived difficulties were some missing foods and meals, the inability to add food items in the program as a user as well as the lack of a mobile app version of the program, while some participants suggested the improvement of the search function in order to be less sensitive to spelling and word order.

## 4 Discussion

Nutrition Data showed comparable dietary intake estimates to interviewer-led 24HRs in men and women with T1D. There were high and statistically significant correlations between Nutrition Data and 24HRs for all intakes, and Bland–Altman analyses showed no systematic differences between the two methods. However, limits of agreement were relatively wide. As for user acceptability, most participants found Nutrition Data easy and fun to use, and would recommend it to others.

Previous studies have also reported fair to good agreement between web-based and conventional dietary records and/or 24HRs (18–21). In a review of different web-based dietary records, there was a ± 17% mean difference for energy and nutrient intake between the web-based and conventional method while correlation coefficients were generally lower or similar to the ones in our study, ranging from 0.37 to 0.87 for energy (kcal/day), 0.31 to 0.82 for carbohydrates (g/day), 0.33 to 0.75 for fat (g/day), and 0.41 to 0.78 for protein intake (g/day) (18). A reason for the stronger correlations observed between Nutrition Data and 24HRs in our study could be that we compared dietary intake from the same 2 days for both methods, using the same program for data entry and nutritional analysis of the 24HRs. Overlapping days were chosen to evaluate short-term intake agreement without increasing participants’ burden too much. It is, however, possible that performing the 24HR on an already registered-in-Nutrition Data day may have increased the participants’ ability to recall intake, decreasing the usual recall bias expected with 24HRs and enhancing agreement with Nutrition Data registrations. Furthermore, using the same program and food database for data entry and nutritional analysis for the 24HRs implies that the same portion size options and food items are available to choose from, which could decrease differences between the two methods and exaggerate their agreement. Similarly, high agreement between methods has been shown in other studies using overlapping days and/or same interface for data entry (19–22).

Limits of agreement between Nutrition Data and 24HRs were relatively wide, indicating low agreement between the methods on an individual level. This is in line with other studies of web-based dietary records compared to conventional paper records or 24HRs performed in adults (19, 21–23). Specifically, for eCa, an electronic food record for tracking of dietary intake that was compared to two telephone 24HRs, limits of agreement ranged from −1,036 to 832 kcal/day for energy and −132.9 to 132.7 g/day for carbohydrates, which were wider than ours (19). The Japanese Internet website dish-based dietary records (WDDRs) were compared against weighed paper dietary records on one day in 161 women and the limits of agreement for energy and carbohydrates were between −261.4 and 333.0 kcal and −25.3 and 27.0 g, respectively (22). In a validation study of DietMatePro, a PDA-based dietary assessment program, dietary intakes from three days of registration in the program were compared against intakes from one 24HR (the last day of the 3-day registration period) and results showed limits of agreement of around ±1700 kcal/day for energy (no data for carbohydrate intake) (21). The online dietary assessment tool myfood24, aimed at the UK adult population with the flexibility to be used both for multiple 24HRs and as a food diary, was compared against two interviewer-led 24HRs of non-overlapping days in a validation study of 212 participants (including comparisons with biomarkers) (23). The study showed that the agreement of myfood24 and 24HRs was moderate (intraclass correlation coefficient range 0.4–0.5 for energy and nutrients) and limits of agreement were −63% to 118% for energy intake (MJ/day) and −70% to 162% for carbohydrate intake (g/day). In practice, the wide limits of agreement between the two methods in our study, indicate that quality controls of individual dietary registrations in Nutrition Data may be needed to ensure the accuracy of the registered data.

Agreement between Nutrition Data and 24HRs in measuring carbohydrate intake was high, and our study participants with T1D found it especially helpful to use Nutrition Data for carbohydrate counting. Carbohydrate counting is recommended for better glycemic control and diet flexibility in people with T1D (3). Nutrition Data allowed also registration of other relevant factors for glycemic control like data on pre- and postmeal glucose, meal insulin doses and physical activity. These factors are crucial for evaluating carbohydrate counting and patients’ carbohydrate-to-insulin ratios (24). Web-based dietary registration programs that include diabetes-specific features could, therefore, be of value for treating patients with T1D.

The strengths of this study include a high participation rate with 42 of the first 44 invited DANCE participants agreeing to participate, a lack of dropouts and minimal missing data. The fact that the validation comprised both men and women of different ages is also a strength. Both weekdays and weekend days were assessed to account for possible differences in food habits (25, 26). In addition, we evaluated Nutrition Data in people with T1D, which are one of the target patient groups in the clinic, instead of using a convenience sample of, for example, student volunteers (19, 27). Regarding the target users, it should be further noted that, although adults with type 2 diabetes (T2D) have, on average, higher age and BMI than the adult T1D population in Sweden (28), they still exhibit similar characteristics as patient groups (e.g., high blood glucose levels, increasing rates of obesity) and some patients with T2D are treated with insulin. Therefore, these findings could be relevant and generalizable to people with T2D that have similar characteristics to our study sample.

This validation study also has limitations including a relatively small sample size, though it should be noted that the study reached the number of participants specified by the power calculation. A selection of participants may have occurred by including a subsample of participants from an RCT with specific inclusion and exclusion criteria. Nevertheless, according to the Swedish Diabetes Registry, our study participants were of similar age, sex, and BMI to the Swedish T1D population in 2022 but had shorter diabetes duration and included more pump users (28). Education level was particularly high in our study, with 62% of our participants having a university degree compared to 42% of the general Swedish population (29). Another limitation is that Nutrition Data was only available in Swedish, which could exclude individuals not speaking the language. Thus, the study participants may be less representable of other adults with T1D without these characteristics, which may limit generalizability of these findings to the general T1D population. Furthermore, it could be argued that following the RCTs dietary interventions with different levels of carbohydrate intake (0–6 months) could have affected the participants’ registrations in Nutrition Data (desirability bias) and possibly the agreement to 24HRs. However, there were no differences in the agreement of Nutrition Data registrations and 24HRs between study period, i.e., 0–6 months vs. 6–12 months when participants ate the diet of their choice. Finally, it was only possible to investigate relative validity and agreement between two subjective methods of dietary assessment in this study and we can therefore not conclude whether Nutrition Data registrations over- or underestimated true intakes of individuals. Subjective methods have their inherent limitations and biases, and the risk of recall bias when using 24HRs should be acknowledged. However, objective methods of dietary assessment, like direct observations and feeding studies, are highly impractical and burdensome for participants, while biomarkers of all nutrients, e.g., total carbohydrate intake, do not exist. Thus, 24HRs are often used as a cost-effective method of comparison and were chosen here to minimize participant burden without affecting their participation to the DANCE study (5). Nutrition Data was also investigated as a possibly more time-efficient alternative to 24HRs for researchers and clinicians, and therefore comparisons of validity between these two methods were of particular interest.

User acceptability and usability of Nutrition Data were in general high, which is often the case, as new technologies are preferred compared to traditional methods (6, 18, 30). However, there were also some problem areas identified that could affect usability, like difficulties in finding some food items, registration time and the lack of a mobile app. The first two problem areas show the need for further training and instructions in order to facilitate registrations. The lack of a mobile app was later solved by program providers. Other factors that may have affected user acceptability and usability include social desirability bias, participants’ technological literacy or previous familiarity with nutrition-tracking software. It is a limitation that these were not assessed in our study.

In conclusion, Nutrition Data had comparable dietary intake estimates to interviewer-led 24HRs in Swedish men and women with T1D. It has the potential to be used by people with T1D (and possibly young normal-weight to overweight people with T2D) for self-monitoring of diet and an as aid for carbohydrate counting, and by health care professionals for assessing patients’ total diet in a time-efficient way. Whether diet registrations and carbohydrate counting with Nutrition Data could affect glycemic control of people with T1D was not in the scope of this study but may be of interest for future research.

## Data Availability

The raw data supporting the conclusions of this article will be made available by the authors, without undue reservation.
