# Supplementary material for: Validation of a web-based dietary assessment program against 24-h recalls in adults with type 1 diabetes
Source: Front Nutr. 2024 Nov 27;11:1395252. doi: 10.3389/fnut.2024.1395252 (PMC11631581; doi:10.3389/fnut.2024.1395252)
Supplement: Supplementary file 1 [file Data_Sheet_1.docx]

Supplementary Material

# Supplementary Data

**Description of 18-item questionnaire assessing usability and user acceptability of Nutrition Data**

Participants were asked to rate their level of agreement or disagreement with 14 statements concerning program usability and satisfaction with program features on a 5-point Likert-type scale. The 15^th^ question asked about the frequency of using Nutrition Data for carbohydrate counting and consequent insulin dosing during the study (5 options available, from “not at all” to “all the time”). The 16^th^ question asked about the timing of the meal registrations in the program, allowing participants to choose multiple options (“many hours before the meal”, “just before eating a meal”, “during a meal”, “just after finishing eating a meal”, “at the end of the day”, “the day after”). The 17^th^ question asked about the average time needed for the participant to register one day’s food and beverage intake in the program (in minutes). The last question was open and optional, asking participants to give feedback about the program’s strengths and weaknesses as well as suggestions for improvements.

**Supplemental Figures and Tables**

**Supplemental Figure 1**. Bland Altman plots showing the difference of the two methods on the y-axis (ND-24HR) and the average of the two methods on the x-axis. Intakes shown: (A) carbohydrates (g/day), (B) sugars (g/day), (C) fiber (g/day), (D) protein (g/day), (E) total fat (g/day), (F) saturated fat (g/day), and (G) (H) alcohol (%TEI and g/day). *ND*; Nutrition Data. *24HR*; 24-hour recalls

| **Supplemental Table 1**. Mean intake (± SD) estimated by means of Nutrition Data registrations and 24-hour food recalls separately for participants during 0-6 (intervention period) and 6-12 months | | | | | | | | | |
| --- | --- | --- | --- | --- | --- | --- | --- | --- | --- |
|  | 0-6 months (intervention period)  n=21 | | | | 6-12 months (diet of choice)  n=21 | | | |  |
| Intake | ND | 24HRs | Diff ND-24HR | *P ^a^* | ND | 24HRs | Diff ND-24HR | *P ^a^* | *P ^b^* diff 0-6 vs  6-12 mo |
| Energy (kcal/d) | 1740 ± 452 | 1784 ± 404 | -44 ± 230 | 0.41 | 1827 ± 414 | 1852 ± 447 | -25 ± 201 | 0.59 | 0.78 |
| Carbohydrate (%TEI) | 34 ± 10 | 34 ± 9 | -0.1 ± 3 | 0.86 | 37 ± 11 | 36 ± 10 | 0.5 ± 4 | 0.79 | 0.88 |
| Fat (%TEI) | 46 ± 10 | 47 ± 9 | -0.3 ± 4 | 0.92 | 44 ± 11 | 45 ± 10 | -0.8 ± 4 | 0.54 | 0.67 |
| Protein (%TEI) | 19 ± 3 | 18 ± 4 | 0.4 ± 2 | 0.70 | 18 ± 5 | 18 ± 6 | 0.4 ± 2 | 0.39 | 0.80 |
| Alcohol (%TEI) | 1 ± 3 | 1 ± 2 | 0 ± 1 | 0.63 | 1 ± 2 | 1 ± 2 | 0 ± 1 | 1.00 | 0.72 |
| Carbohydrate (g/d) | 137 ± 45 | 144 ± 46 | -7 ± 20 | 0.22 | 158 ± 54 | 158 ± 55 | 0.2 ± 17 | 0.83 | 0.24 |
| Sugars (g/d) | 42 ± 17 | 41 ± 16 | 0.1 ± 9 | 0.97 | 51 ± 25 | 52 ± 25 | -1.6 ± 11 | 0.46 | 0.62 |
| Fibre (g/d) | 21 ± 8 | 20 ± 6 | 0.6 ± 4 | 0.82 | 21 ± 8 | 21 ± 7 | -0.3 ± 4 | 0.82 | 0.50 |
| Fat (g/d) | 91 ± 33 | 94 ± 29 | -3 ± 16 | 0.54 | 91 ± 35 | 93 ± 35 | -2.5 ± 19 | 0.68 | 0.94 |
| Saturated fat (g/d) | 29 ± 11 | 30 ± 11 | -1 ± 6 | 0.33 | 31 ± 13 | 31 ± 14 | 0.1 ± 7 | 0.44 | 0.21 |
| Protein (g/d) | 80 ± 24 | 81 ± 25 | -0.4 ±11 | 1.00 | 81 ± 27 | 80 ± 28 | 1 ± 10 | 0.73 | 0.61 |
| Alcohol (g/d) | 2 ± 6 | 2 ± 6 | -0.1 ± 2 | 1.00 | 2 ± 5 | 2 ± 4 | 0.1 ± 3 | 0.83 | 0.82 |
| *^a^ P* for differences between ND and 24-hour recall mean intakes within each study period (intervention: 0-6 mo, no intervention: 6-12 mo) using Wilcoxon matched-pairs signed-rank test  *^b^ P* for intake differences (ND-24HR) between study periods (0-6 vs 6-12 mo) using Wilcoxon rank sum test or un-paired t-test  *ND*; Nutrition Data. *24HRs*; 24-hour recalls. *Diff*; difference. | | | | | | | | | |

| **Supplemental table 2**. Correlations between Nutrition Data registrations and 24-hour recalls for total sample and per study period (0-6 and 6-12 months). | | | |
| --- | --- | --- | --- |
|  | **Spearman’s rho** | | |
|  | N=42 all | 0-6 months (intervention period)  n=21 | 6-12 months  (diet of choice)  n=21 |
| Energy kcal/day | 0.79 | 0.78 | 0.78 |
| Carbs % TEI | 0.94 | 0.91 | 0.95 |
| Carbs g/day | 0.93 | 0.91 | 0.92 |
| Of which, sugars g/day | 0.87 | 0.90 | 0.82 |
| Fiber g/day | 0.83 | 0.92 | 0.80 |
| Fat %TEI | 0.90 | 0.89 | 0.92 |
| Fat g/day | 0.85 | 0.86 | 0.85 |
| Saturated fat g/day | 0.89 | 0.88 | 0.90 |
| Protein % TEI | 0.84 | 0.79 | 0.84 |
| Protein g/day | 0.86 | 0.85 | 0.89 |
| Alcohol %TEI | 0.88 | 0.89 | 0.87 |
| Alcohol g/day | 0.90 | 0.99 | 0.83 |
| *P* < 0.001 for all coefficients | | | |

| **Supplemental table 3**. Quality evaluation of dietary assessment of Nutrition Data *^a^* | | |  |
| --- | --- | --- | --- |
|  |  | Points | |
| Dietary assessment method | Food record |  | |
| Validation? | Yes |  | |
| Validation method | 24-hour recall |  | |
| 1. Validation sample | Validated in same population as for intervention study | 0.5 | |
| 2. Statistics to assess validity | Test mean + correlation + Bland Altman plots + multiple methods used | 2.5 | |
| 3. Data collection | Food records are self-administered | 0 | |
| 4. Data analysis | Analysed by a trained person | 0.5 | |
| 5. Scoring method | For nutrient calculations -relevant nutrient databases reported | 1 | |
| 6. Days recall | 2 | 0 | |
| 7. Use multiple pass & aids | Aids/ prompts/ weight used for portion size estimation; Analysis by a trained person | 0.5 | |
| Total Score (max score 8) |  | 5 | |
| Overall quality |  | Medium | |
| *^a^* According to EURICA tool by Wang T, Siopis G, Wong HY, Allman-Farinelli M. Poor quality of dietary assessment in randomized controlled trials of nutritional interventions for type 2 diabetes may affect outcome conclusions: A systematic review and meta-analysis. Nutrition. 2022 Feb;94:111498. doi: 10.1016/j.nut.2021.111498. | | | |
